# Assessing the Antioxidant Benefits of Topical Carvacrol and Magnolol Periodontal Hydrogel Therapy in Periodontitis Associated with Diabetes in Wistar Rats

**DOI:** 10.3390/dj11120284

**Published:** 2023-12-08

**Authors:** Georgiana Ioana Potra Cicalău, Gabriela Ciavoi, Ioana Scrobotă, Andreea Olivia Marcu, Ioana Romanul, Eleonora Marian, Laura Grațiela Vicaș, Mariana Ganea

**Affiliations:** 1 Department of Dental Medicine, Faculty of Medicine and Pharmacy, University of Oradea, 1st Decembrie Street, 410073 Oradea, Romania; cicalau.georgiana@uoradea.ro (G.I.P.C.); gciavoi@uoradea.ro (G.C.); ioana_romanul@uoradea.ro (I.R.); 2 Preclinics Department, Faculty of Medicine and Pharmacy, University of Oradea, 410073 Oradea, Romania; 3 Department of Pharmacy, Faculty of Medicine and Pharmacy, University of Oradea, 1st Decembrie Street, 410073 Oradea, Romania; emarian@uoradea.ro (E.M.); lvicas@uoradea.ro (L.G.V.); mganea@uoradea.ro (M.G.)

**Keywords:** periodontitis, diabetes, oxidative stress, carvacrol, magnolol

## Abstract

It is well recognized that oxidative stress contributes to chronic stress-induced cytotoxicity, which is a major factor in the progression of many diseases, including periodontitis and diabetes. Formulas based on natural extracts with antioxidant properties are alternative treatment perspectives in the management of such diseases. The aim of our study was to assess how carvacrol and magnolol influence periodontitis associated with diabetes in Wistar rats. Ninety Wistar rats were distributed in nine groups: I—control group; II—diabetes group (D); III—periodontitis group (P); IV—periodontitis and diabetes group (PD); V—periodontitis and diabetes with vehicle alone (PDV); VI—periodontitis and diabetes treated with carvacrol (PDC); VII—periodontitis and diabetes treated with magnolol (PDM); VIII—periodontitis and diabetes treated with carvacrol and magnolol (PDCM); IX—healthy group with vehicle alone (CV). Blood malondialdehyde (MDA) levels and catalase activity levels (CAT) were measured as indicators of oxidative stress and antioxidant capacity, respectively. Where diabetes and periodontitis were induced, MDA was augmented and CAT was depleted significantly. Whether given alone (PDM) or in combination with carvacrol (PDCM), magnolol significantly decreased MDA. Between the PDM group and the PDCM group, there were no notable differences. In Wistar rats with periodontitis related to diabetes, topical use of hydrogels containing magnolol, either alone or in combination with carvacrol, may reduce oxidative stress.

## 1. Introduction

Several studies have shown the bidirectional relationship between periodontal disease and diabetes [1,2,3,4]. Both pathologies are highly prevalent worldwide, but the mechanisms linking them are not fully understood [5]. According to the studies of Sanz et al., elevations in oxidative stress as well as in important cytokines implicated in inflammatory signaling pathways, are among the mechanistic linkages between diabetes and periodontitis [1]. Mohamed et al. consider that chronic periodontitis is associated with disturbance of the local expressions of biomarkers related to the onset of type 2 diabetes and its medical complications in gingival crevicular fluid [2]. Casanova et al. highlights that diabetes and periodontitis are chronic conditions that have a known reciprocal association, so that patients with diabetes have shown improvements in glycaemic control after receiving periodontal treatment, with HbA1c levels decreasing by about 0.4% [3,4].

Periodontal involvement in systemic conditions is also described. Relationship between cardiovascular pathology and periodontal illnesses due to systemic inflammation with increased circulating cytokines and mediators, direct infection, and cross-reactivity between bacterial antigens and self-antigens is stated by Seymour et al. [6] and by Pardo et. al., respectively [7].

Still, diabetes remains the most significant systemic disease found in the pathological personal history of patients arriving at the dental office, according to research on the incidence of diabetes mellitus and oral disorders [8]. Understanding the interrelationship between these conditions could improve their screening and management, bringing important benefits to patients. Epidemiological studies highlight diabetes as a major risk factor for periodontal disease, the risk of developing periodontal diseases being greater the weaker the metabolic control [9,10]. In a review on the interrelation between diabetes and periodontitis, Stohr et al. highlighted the importance of screening patients with diabetes or periodontitis in regard to the risk of their association [11].

All aerobic cells are equipped with a protection system that generates reactive oxygen species (ROS) (e.g., superoxide radical (O_2_^•−^), hydrogen peroxide (H_2_O_2_), hydroxyl free radical (OH^•^), peroxinitrite (ONOO^−^)) in order to cope with microorganisms and intracellular cell signaling. The organism antioxidant capacity counteracts the accumulation of ROS via proteasome and autophagy. When a disequilibrium between pro and antioxidants appears, either by increased ROS production or decreased antioxidant capacity, oxidative stress is installed and results in damage to lipids, nucleic acids, and proteins [12].

The accumulation of ROS is considered to be implicated in the pathogenesis of numerous diseases since almost all inflammatory conditions are related to oxidative stress [13]. Oxidative stress may be involved in chronic stress-induced cytotoxicity, playing a critical role in the aggravation of periodontitis and diabetes [14]. Diabetes induces a state of oxidative stress that disrupts the balance between ROS production and inactivation [15]. In diabetes, there can be activated biochemical pathways like glucose auto-oxidation, polyols pathway, prostaglandins synthesis, and protein glycation. These mechanisms, strictly related to hyperglycemia, increase the production of circulatory ROS [16]. At the periodontal level, evidence linking ROS to pathological connective tissue destruction during periodontitis is based on the presence of neutrophil infiltration as a major event in the host response to bacterial invasion [17]. Stimulated by pathogens in the dental biofilm, neutrophils become the most important source of ROS in periodontitis [18]. Several studies attested increased oxidative stress activity in peripheral blood neutrophils of periodontitis patients compared to controls [18]. Therefore, decreasing local periodontal oxidative stress by using antibacterial, anti-inflammatory agents could improve both local and general status.

ROS generate the process of lipid peroxidation [19], whereby oxidants attack lipids containing carbon-carbon double bonds, especially polyunsaturated fatty acids [20]. Malondialdehyde (MDA) is one of the end products of the peroxidation of polyunsaturated fatty acids, and the increase of free radicals causes the overproduction of MDA [5]. MDA has a high capacity to react with multiple biomolecules, such as proteins or DNA, leading to the formation of adducts and excessive production of MDA, which has been associated with various pathological conditions [21]. The level of MDA is commonly assessed as a marker of oxidative stress [19,21].

The antioxidant defense system includes both endogenous, enzymatic, and non-enzymatic antioxidants, such as superoxide dismutase, catalase, glutathione peroxidase, and glutathione, as well as exogenous antioxidants, the food being their main source. Regarding exogenous antioxidants, carotenoids (lycopene, lutein, zeaxanthin, α- and β-carotene, β-cryptoxanthin), vitamin E (α- and γ-tocopherol), and polyphenols are known [22].

First-line defense antioxidants include catalase, a key detoxifying enzyme present in the peroxisomes of all aerobic cells. Catalase is a powerful oxidative agent whose primary function is to break down H_2_O_2_ into water and oxygen, which prevents cells from developing oxidative stress [21,23].

Supplementation with natural antioxidants has been reported to enhance the performance of the human body during exposure to stressors [24]. Among them, carvacrol has strong antioxidant properties and a protective effect against free radicals and has been found effective in preventing and inhibiting cardiac, liver, and metabolic diseases [25,26,27,28]. Magnolol scavenges OH^•^, ONOO^−^ [29] and H_2_O_2_ [30], suppressing ROS generation in the same pathologies [25].

This experiment aimed to evaluate in vivo the antioxidant effect of carvacrol and magnolol on experimental animals with periodontitis and diabetes by evaluating some markers involved in oxidative stress (MDA) and antioxidant defense capacity (CAT).

The present research tested the hypothesis that topical periodontal application of hydrogels containing carvacrol and magnolol may modulate the oxidative stress in periodontitis associated with diabetes.

## 2. Materials and Methods

### 2.1. In Vitro Experiment Design

#### 2.1.1. Materials and Drugs Used

All chemicals and reagents used have a high degree of purity. The 2,2′-azino-bis (3-ethylbenzthiazoline-6-sulphonic acid) (ABTS), 2,2-diphenyl-2-picryl-hydrazyl (DPPH) and potassium persulfate used were purchased from Sigma-Aldrich^®^ Chemie GmbH, Taufkichen, Germany. Streptozotocin and the natural extracts, carvacrol and magnolol, were purchased from Sigma-Aldrich^®^, Inc., St. Louis, MO, USA. PBS (phosphate buffered saline (pH = 7.4)), etanol, polietilenglicol and carbopol 940 were obtained from S.C. VITAMAR IMPORT EXPORT SRL, Bucharest, Romania. Double-distilled water was obtained using a Milli-Q system (Millipore, Bedford, MA, USA).

#### 2.1.2. In Vitro Determination of Antioxidant Activity of Carvacrol and Magnolol

To evaluate the free radical scavenging activity of different concentrations of magnolol and carvacrol, both ABTS and DPPH tests were performed.

The method of testing the antiradical capacity with the application of ABTS is known and used on a large scale to determine the antioxidant activity of substances regardless of their nature. Thus, the antioxidant activity of both pure substances and antioxidant complexes were determined [31].

The 2,2′-azino-bis (3-ethylbenzthiazoline-6-sulphonic acid) (ABTS°+) radical cation was obtained by reacting the stock solution of 7 mM ABTS with 2.45 mM potassium persulfate for 16 h. Then, the ABTS solution was diluted with phosphate buffered saline (PBS) (pH = 7.4) until an absorbance of 0.7 ± 0.02 was obtained, read using a PG Instruments T70+ spectrophotometer at 734 nm. Then, 10 μL of the samples of different concentrations were added to 3.0 mL of ABTS, shaken, and kept at room temperature and in the dark for 6 min, then their absorbances were read at 734 nm. The tests were performed in triplicate. The total equivalent antioxidant capacity was calculated with the equation:

$$Scavenging\;effect\;\left(\%\right)=(1-\frac{A_{sample}}{A_{control}})\times 100$$

where *A_sample_* is the absorbance of the sample and *A_control_* is the absorbance of the control.

Free radical scavenging activity was measured using 2,2-diphenyl-2-picryl-hydrazyl (DPPH°) [32]. For this method, there are studies that have shown that the solvent used in the step of extracting the hydrogen atom from the phenolic O-H bond is important [33]. There are authors who state that the ionization of the phenolic hydroxyl, thus the extraction of the hydrogen atom from the phenolic hydroxyl, is a slow process, which could take place faster if one were to work with strong solvents (alcohol solvents) that form hydrogen bonds, such as ethanol and methanol [34]. In the present work, the solvent used was ethanol.

To begin, 2.9 mL of DPPH solution was added to 0.1 mL of the samples of different concentrations. Then, after vortexing, they were kept in the dark at room temperature, and then their absorbances were measured at 517 nm, using a spectrophotometer PG Instruments T70+. Double-distilled water was used as a control. The tests were performed in triplicate. The inhibition percentage was calculated using the equation:

$$Inhibition\;\left(\%\right)=\frac{{A_{control}-A}_{sample}}{A_{control}}\times 100$$

where: *A_control_* is the absorbance of the control and *A_sample_* is the absorbance of the sample.

#### 2.1.3. Preparation of Hydrogels

**Figure 1:**
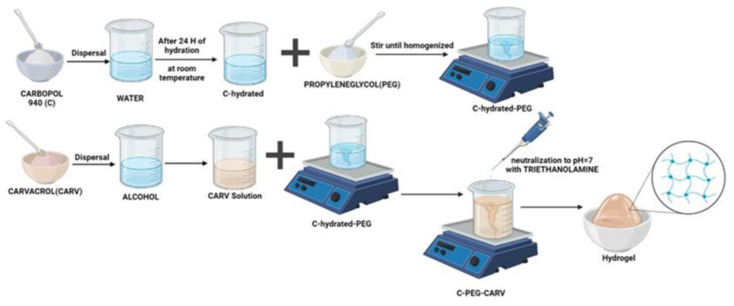
Schematic representation of the preparation of carvacrol hydrogels.

When preparing hydrophilic gels, the properties of all components were taken into account, so the consistency agent (carbopol 940) was dispersed in the vehicle (water) and left to hydrate for 24 h. The dispersing agent (propylene glycol) was then added and stirred until homogeneous. Then the therapeutic agent or active ingredient (carvacrol and magnolol respectively) was added, which was previously dispersed in alcohol. The mixture was brought to a neutral pH by neutralization with triethanolamine, and it was supplemented with double-distilled water, stirring continuously, until the formation of the hydrogel (Figure 1). Magnolol hydrogels are also prepared in the same way. The preparation formulas are shown in Table 1, as we previously mentioned in another article [35].

#### 2.1.4. Release of Carvacrol and Magnolol from Hydrogels

To qualitatively and quantitatively evaluate the hydrogels, we measured the release of carvacrol and magnolol, respectively, from the hydrogels [36]. A six cell Franz diffusion system (Microette-Hanson system, model 57-6AS9, Copley Scientific Ltd., Nottingham, UK) was used. Each receptor chamber has a diffusion surface of 1.767 cm^2^ and a volume of 6.5 mL and was filled with PBS (pH 7.4) or PBS mixed with freshly prepared 30% ethanol. Synthetic polysulfone membranes with a diameter of 25 mm and pores of 0.45 m were used (Tuffryn^®^, PALL Life Sciences HT-450, lot T72556), which were hydrated for 30 min by immersion in the receptor medium before use. The sample consists of 0.500 g of hydrogel that is placed in the capsule of the diffusion cell. The system temperature was maintained at 32 ± 1 °C, and the receptor medium was continuously stirred (600 rpm) using a magnetic stirrer. For the determination, 0.5 mL of the receptor solution was taken at different time intervals (from 15 min up to 120 min), and the amount of carvacrol or magnolol released was determined using a UV-VIS spectrophotometer, PG Instruments T70+, the reading being performed at 275 nm for carvacrol and 293 nm for magnolol.

### 2.2. In Vivo Experiment Design

#### 2.2.1. The Motivation for Choosing the Experimental Model

Among the small laboratory animals, the rat is the most extensively studied in the pathogenesis of periodontitis. The induction of periodontitis and diabetes requires complete biological systems for simulation under conditions effectively comparable to human subjects, so alternatives such as tissue or cell systems cannot be used in this study. Cell cultures are used in preclinical research, but have limitations, not being characteristic of ongoing physiological processes [37]. The experimental induction of inflammation in rats has been shown to be the closest to the characteristics of human inflammation [38,39]. In experimental research, the most widely used breeds of rats are the Wistar breed and the Spraque-Dawley breed [40,41].

Previous studies have demonstrated that in vivo models are essential for reproducibility under experimental conditions of periodontitis and diabetes and also for studying therapeutic efficacy at the biochemical level. Natural extracts’ effects can be, therefore, tested at a clinical and paraclinical level in the mentioned pathologies [42,43].

#### 2.2.2. Distribution of Animals Used in the Experimental Model

The experiment was carried out at the Biobase of the Physiology Department of the “Iuliu Haţieganu” University of Medicine and Pharmacy in Cluj-Napoca. Following the evaluation of the protocol, the experimental research was approved by the Ethics Committee of the “Iuliu Hațieganu” University of Medicine and Pharmacy in Cluj-Napoca and received favorable opinion no. 316 of 15 September 2021.

The present study is a parallel prospective placebo-controlled experimental analytical study. The in vivo study was carried out on 90 white, male Wistar albino rats (Rattus norvegicus) aged 8 weeks, with an average weight between 180 and 220 g. The animals used come from the Biobase of the Department of Physiology of the University of Medicine and Pharmacy “Iuliu Haţieganu” from Cluj-Napoca, Romania.

The animals were kept in separate polypropylene boxes, in a temperature-controlled environment (21 ± 2 °C), exposed to a 12-h light-dark cycle, and 70 ± 4% humidity. Animals were housed under normal laboratory conditions and provided with standardized food and water ad libitum. The bed was represented by aseptic autoclaved wood fragments, enriched with “environmental enrichment” products. The rats were acclimatized for one week before starting the experiment. The health status of the animals was checked periodically throughout the experiment. At the end of the project the animals were not relocated.

#### 2.2.3. Experimental Induction of Diabetes Mellitus

Diabetes was induced by a single intraperitoneal dose of streptozotocin (60 mg/kg) (Sigma-Aldrich^®^, St. Louis, MO, USA). Before the induction of diabetes, rats’ weights were registered, and the blood glucose values were assayed from samples collected from the caudal vein of the animals. In the final three days of the study, all rats, except the control groups (C, CV), were given intraperitoneal injections of streptozotocin, separated by 72 h, in order to develop diabetes mellitus [44]. Through beta cell destruction, streptozotocin causes diabetes in three days [45,46]. Diabetes was confirmed three days after the streptozotocin injection when the blood glucose value was recorded again, following the doubling of the initial values [46,47,48]. The average values of blood glucose in the groups of rats in which diabetes was induced were between 326.8 ± 11.27 mg/dL and 331.4 ± 7.97 mg, while in the case of the other groups (C, P, CV) average blood glucose values ranged between 117.3 ± 4.37 mg/dL and 118.4 ± 4.94 mg/dL.

#### 2.2.4. Experimental Induction of Periodontitis

After the rats were anesthetized by an intramuscular injection of ketamine (90 mg/kg) and xylazine (10 mg/kg), a surgical ligature was applied in the gingival sulcus of the left mandibular first molar The 0.8 mm diameter stainless steel and chromium orthodontic wire ligatures were kept in place for three consecutive months, favoring the adhesion of dental plaque, gingival inflammation, and periodontitis. At the end of the three months, an examination was conducted on all surfaces of the ligatured teeth by moving the dental probe on all surfaces of the tooth and probing in six sites: three on the buccal side and three on the oral side (mesial, central, and distal of the tooth). The deepest pocket found had an average pocket depth of 3.6 mm. Before starting treatment with periodontal hydrogels, orthodontic ligatures were removed [49,50,51,52].

#### 2.2.5. Experimental Design

The animals were randomly distributed into nine groups (*n* = 10):

Group I—healthy group (C);

Group II—rats with induced diabetes (D);

Group III—rats with induced periodontitis (P);

Group IV—rats with induced periodontitis and diabetes (PD);

Group V—rats with induced periodontitis and diabetes, vehicle hydrogel treatment (PDV);

Group VI—rats with induced periodontitis and diabetes treated with carvacrol (PDC);

Group VII—rats with induced periodontitis and diabetes treated with magnolol (PDM);

Group VIII—rats with induced periodontitis and diabetes treated with carvacrol and magnolol (PDCM);

Group IX—healthy group, treatment with vehicle gel (CV).

After diabetes and periodontitis were installed, 400 µg/mL of carvacrol and 25 µg/mL of magnolol incorporated in bioadhesive hydrogels using carbopol 1% [35] were topically applied in the gingival sulcus and the adjacent oral mucosa. A syringe with a blunt tip was used at the level of the dental package of the molar with ligature, then dispersed evenly with a spatula on the vestibular and lingual surfaces of the tooth [53,54]. The hydrogel was applied two times a day; 1 g hydrogel was administered in the morning and 1 g hydrogel in the evening for a period of 1 month, until the end of the experiment. In the case of the PDCM group, the carvacrol hydrogel was applied in the morning and the magnolol hydrogel in the evening. After applying the hydrogel, the animals were restrained from food and water for 1 h for a better absorption at the gingival level.

Topical administration of carvacrol and magnolol hydrogels is not thought to cause harm [55,56,57,58,59]. The reduction of possible adverse reactions as a result of the administration of plant extracts was counteracted by continuous monitoring of the animals’ condition.

At the end of the periodontal treatment, blood samples were collected under general anesthesia with ketamine and xylazine.

### 2.3. Blood Sample Collection and Analyses

Blood samples were collected from the retro-orbital sinus. To begin, 5 mL of blood was collected from each rat to examine oxidative stress and antioxidant defense. The serum was separated by centrifugation at 3000 rpm for 15 min. Samples were centrifuged at a low temperature of 4 °C, and the supernatant was sealed and stored at −85 °C until analysis. MDA (nmol/mL) was measured from serum as a parameter of oxidative stress and CAT (U/mg protein) from erythrocyte lysate as a parameter of antioxidant defense.

The biochemical markers were dosed in the Oxidative Stress Research Laboratory of the Physiology Department of the “Iuliu Hațieganu” University of Medicine and Pharmacy in Cluj-Napoca.

At the end of the experiment, all animals were euthanized under analgo-sedation conditions. Euthanasia was performed with a triple dose of ketamine and xylazine, followed by cervical dislocation. The methods used were in accordance with the provisions of art. 5 para. (5) lit. a) from Law no. 3/2014 on the protection of animals used for scientific purposes, with subsequent amendments, and no exemptions were requested.

### 2.4. Determination of the Lipid Peroxidation Indicator—MDA

The indicator of lipid peroxidation, MDA, was determined by the spectrofluorimetric method described by Conti et al. (1991) [60]. The method was based on the fact that the MDA resulting in this process forms a fluorescent adduct with 2-thiobarbituric acid (TBA). For MDA assay, the serum sample was boiled for 1 h with a solution of 10 mM TBA in 75 mM K_2_HPO_4_ at pH 3. After quenching, the reaction product was extracted into n-butanol. The concentration was determined in the organic phase after its separation by centrifugation. Emission intensity measurement was performed at 534 nm with a Perkin–Elmer spectrofluorometer, by a synchronous fluorescence technique, at an excitation-emission wavelength difference of 14 nm. The concentration of MDA was established on the basis of a calibration curve made with known concentrations of MDA processed in the same way. Serum concentration values were expressed in nmol/mL [60].

### 2.5. Determination of the Indicator of Antioxidant Defense—CAT

The antioxidant defense indicator, CAT, was determined by the enzymatic method described by Pippenger et al. (1998) [61]. CAT activity was measured in a reaction mixture containing 10 mM hydrogen peroxide in 50 mM potassium phosphate buffer at pH 7.4. The amount of enzyme that produced a decrease in absorbance of 0.43 at 25 °C per minute at 240 nm in this system was defined as one unit of catalase activity. CAT activity was expressed as U/mg protein [61].

### 2.6. Statistical Analysis

The statistical analysis was performed in the SPSS24 Software (version 24, Armonk, New York, NY, USA) dedicated to statistical processing. To test whether there was a significant impact on the groups of rats following the application of the gels, the ANOVA test was applied, with the Scheffe test to test for significant differences between pairs of groups. The impact of placebo treatment with vehicle gel administered to the PDV group was tested using the paired Student’s *t*-test. The level of significance considered is *p* < 0.05. If not, it is specified.

## 3. Results

We assayed the antioxidant capacity of carvacrol and magnolol using ABTS and DPPH tests. At ABTS test application, different concentrations of carvacrol and magnolol resulted different scavenging activities of the hydrogels (Table 2).

DPPH, another method we applied, consists of a reaction mechanism of abstraction of a hydrogen atom from a donor phenol and its coupling to the DPPH radical reagent with the formation of a phenoxy radical and DPPH-H. The calculation formula was identical to that of the ABTS test (Table 3).

In vitro release profiles of formulations containing carvacrol or magnolol were investigated with the Franz diffusion method. Samples were taken from the receiver every 15 min for up to 2 h. The permeation profiles of the active ingredients showed dependence on the concentration of viscosity-increasing agent (carbopol 940) (Table 4).

**Figure 2:**
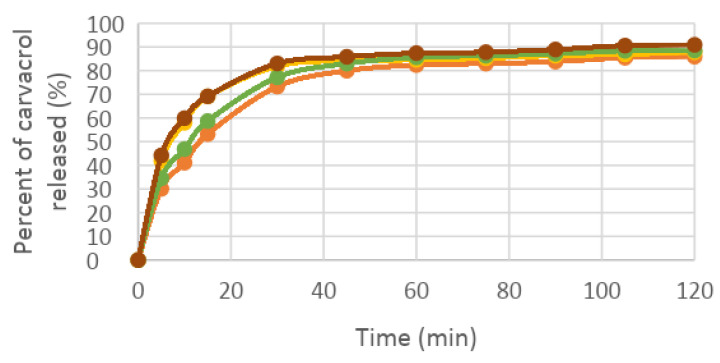
The percentage of carvacrol released in phosphate tampon: orange—hydrogel 1, yellow—hydrogel 2 and in mixture phosphate tampon-alcohol: green—hydrogel 1, brown—hydrogel 2.

**Figure 3:**
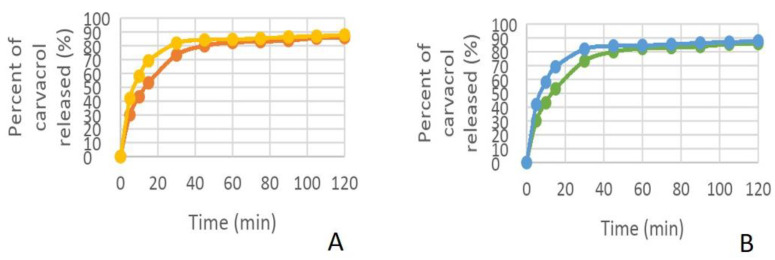
The percentage of carvacrol released. (**A**) in phosphate tampon: orange—hydrogel 1, yellow—hydrogel 2; (**B**) in mixture phosphate tampon-alcohol: green—hydrogel 1, blue—hydrogel 2.

**Figure 4:**
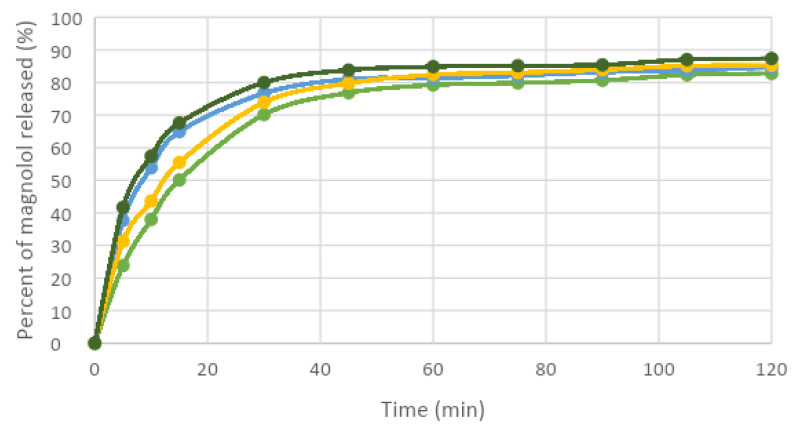
The percentage of magnolol released in phosphate tampon: green—hydrogel 3, blue—hydrogel 4 and in mixture phosphate tampon-alcohol: yellow—hydrogel 3, dark green—hydrogel 4.

**Figure 5:**
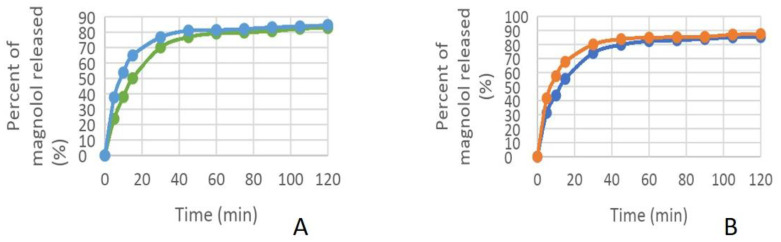
Percentage of magnolol released; (**A**) in phosphate tampon: green—hydrogel 3, blue—hydrogel 4; (**B**) in mixture phosphate tampon-alcohol: blue—hydrogel 3, orange—hydrogel 4. When attempting to induce the diabetes, after streptozotocin administration we obtained a median value of glycemia of 300 ± 50 mg/dL. The values were not significantly modified after the hydrogels’ treatment.

The in vitro permeation profiles of carvacrol and magnolol through the membrane impregnated with the receptor solution was also assayed (Figure 2, Figure 3, Figure 4 and Figure 5).

At the end of the experiment, based on the results recorded from the blood level, the descriptive and comparative statistical analysis of the nine groups of rats was performed.

**Figure 6:**
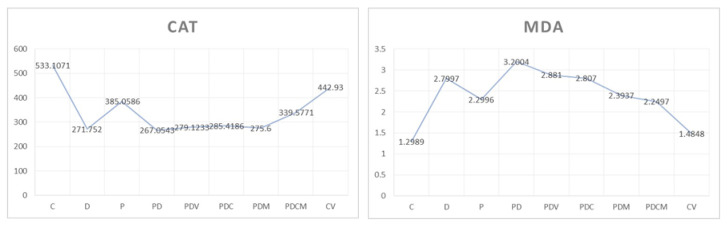
Mean blood MDA and CAT values for all groups of rats.

Regarding the values of the MDA and CAT markers, we compared the results in C-D-P-PD, PD-PDV-PDC-PDM-PDCM, and C-CV groups (Figure 6).

The ANOVA test for MDA and CAT variables in C, D, P, and PD groups resulted in significant differences in the mean values of MDA and CAT in the four groups of rats (*p* = 0.000) (Table 5).

To identify pairs of lots that show significant differences, the Scheffe test is applied (Table 6).

After the hydrogel application, when applying the ANOVA test for the MDA and CAT in PD, PDV, PDC, PDM, and PDCM group, significant differences are observed in the MDA values in the five groups of rats (*p* = 0.000), but there are no significant differences for the CAT values (*p* = 0.052) (Table 7).

To identify pairs of lots that show significant differences, the Scheffe test was applied (Table 8).

We also studied whether the gel used as a vehicle is involved in lipid peroxidation or antioxidant defense. A comparison was made between the C and CV groups to see if there were significant differences in MDA and CAT markers. Baseline values (group C) and values after hydrogel application (group CV) were compared. To determine whether the gel base has a significant effect, the Paired *t*-Student test was used (Table 9).

## 4. Disscusion

In our research, the induction of periodontitis and the diabetes in Wistar rats resulted in increased values of MDA, an indicator of oxidative stress, and decreased values of CAT, an indicator of the antioxidant capacity, measured in the blood of the experimental animals. To counteract the oxidative stress, we used periodontal hydrogels in which we incorporated carvacrol and magnolol.

For the induction of periodontitis, we used orthodontic wires placed around the cervical region of the second lower molars of the rats to promote the accumulation of the bacterial plaque and the instalation of periodontitis. Ligatures-induced periodontitis in rats is a frequently used method. Molecular alteration in this experimental model are the same with the ones that humans develop in periodontitis. Clinically, ligature-induced periodontitis produces the distruction of the gingival atachement, the apical migration of the jonctional epithelium, and bone resorbtion [62,63].

Diabetes was induced with streprozotocine. Streprozotocine administration in rats results in structural, functional, and biochemical modifications similar to those present in patients with diabetes [64]. The pathogenetic mechanism is based on the reduction of nicotinamide adenine dinucleotide in the pancreatic Langerhans beta cells by streprozotocine, followed by histopatologic events that mediate diabetes instalation [46].

MDA values increased significantly in groups D, P, PD vs. C (*p* < 0.05), implicating oxidative stress in the pathogenesis of these diseases [65]. Similar observations were made in other studies [47,66,67,68]. Comparing periodontitis rats (P group) and periodontitis with diabetes rats (PD group), we obtained MDA values significantly raised in PD group. The accumulation of oxidative stress in the case of the association between the diseases could explain our outcomes. Other researchers recently observed that simultaneous induction of periodontitis and diabetes synergistic aggravated the local and general oxidative alterations [39]. Their conclusion was supported by the fact that periodontitis was more severe when associated with diabetes [39].

When evaluating the antioxidant defense, we determined significantly lower CAT levels in the P, D, and DP groups compared with the control group (C) (*p* < 0.05). This result could be explained by the depletion of the antioxidant capacity in the attempt to counteract the oxidative stress [51,69].

In the case of diabetes (group D) and diabetes and periodontitis (PD), the antioxidant capacity was more altered, with CAT levels being significantly lowered compared with those registered in the periodontitis group (P) (*p* < 0.05). Our results support other studies in which diabetes reduced the antioxidant defense [70]. Diabetes type 2 hyperglycemia reduces the production and activity of many antioxidant enzymes, including CAT, probably by glycation mediation. Moreover, in diabetes, the antioxidant nonenzymatic defense (vitamin C, E, A) is also diminished, amplifying the oxidative stress [71].

To counteract the oxidative stress implicated in the pathogenesis of periodontitis and diabetes, we used periodontal gels in which we incorporated carvacrol and magnolol.

In establishing the composition of the hydrogels and testing the performance of hydrogels, the concentration of carbopol in the formula influenced the release of the active ingredient.

Thus, the higher the amount of carbopol used in the formulation of hydrogels, the slower was the release of the active ingredient from the hydrogels.

Another factor influencing the release of the active ingredient from the hydrogel was the alcohol concentration. The presence of alcohol in the release medium stimulated the faster release of the active ingredient from the hydrogel.

The IC50 was obtained for a concentration of 0.214 mg/mL for carvacrol and 0.014 mg/mL for magnolol [72,73,74,75].

The antioxidant capacity of carvacrol was demonstrated in other in vivo and in vitro studies as well. Carvacrol was found to inhibit the oxidation due to its –OH group bonded to the aromatic ring [28,76], to eliminate free radicals and ROS [27,77,78], enhance the production of CAT thereby preventing the tissue alterations resulted from chronic stress [79,80,81]. A previous study also supports our findings that carvacrol could reduce MDA and increase CAT, therefore sustaining carvacrol reducing oxidative stress [82].

In exclusive administration of carvacrol (PDC group), the present research revealed a non-significant decrease in MDA values and a non-significant increase in circulating CAT values when compared to the PD group. We consider that a higher animal number in a future study, or increased carvacrol concentration in the gels, could result in statistically significant results.

Magnolol was less studied than carvacrol and more studied in relation with diabetes and its complications than periodontitis. Magnolol was found to have antioxidant and anti-inflammatory properties via inhibition of AGE, glycation end products that upregulate the synthesis of proinflammatory mediators as TNF-a and IL-6. AGE generates ROS that seem to contribute to the vascular lesions implicated in different complications of diabetes [83,84,85]. CAT was also augmented by oral administration of magnolol in an in vivo diabetes experiment [86]. Recently, magnolol was reported to reduce ROS production in an in vitro diabetic periodontitis model [87].

In our study, the single application of magnolol hydrogel in rats with diabetes and periodontitis (PDM group) demonstrated a significant decrease in MDA values (*p* < 0.05). and a non-significant increase in circulating CAT values when compared to the PD group.

By comparing the mean values of the MDA marker in the PDC and PDM groups, we found a greater decrease in this marker after magnolol administration. Regarding the mean values of the CAT marker, the comparison between the same groups identified a better increase of this marker after the administration of carvacrol. To decide whether to accept or reject the insignificant changes found, they must be investigated in larger groups of rats. The fact that magnolol is more effective on MDA and carvacrol on CAT might determine a better antioxidant effect in the case of combined treatment.

In the situation where we applied both extracts (PDCM group), we obtained significantly better results compared to the independent administration of carvacrol (PDC group). This may be due to the better efficacy of carvacrol on CAT and magnolol on MDA, thus demonstrating a synergistic relationship.

The association of carvacrol with magnolol (PDCM group) demonstrated a significant decrease in MDA values (*p* < 0.05) and a non-significant increase in CAT values in the blood of rats with periodontitis associated with diabetes mellitus when compared to the PD group. It is possible that significantly improved general antioxidant defense would be evident after a longer period of local gels application.

In the pair of groups C-CV, there are no significant differences in the level of the MDA marker (*p* = 0.211) and in the level of the CAT marker (*p* = 0.054), which means that the placebo-administered vehicle gel was not involved in the production of oxidative stress and could be used as a vehicle for the incorporation of various natural extracts. Our results show that the association of the two extracts has a potentiated effect in reducing lipid peroxidation.

We have not identified, in the specialized literature, studies comparing the associated therapeutic effect of carvacrol with magnolol. To our knowledge, the present research studies the antioxidant effect of the combined treatment of the two extracts on periodontitis associated with diabetes mellitus for the first time.

Since both carvacrol and magnolol have antibacterial activity on the periodontal biofilm by exerting their action on microorganism like Aggregatibacter actinomycetemcomitan, Porphyromonas gingivalis, Fusobacterium nucleatum, Prevotella intermedia, or Micrococcus luteus [25,56,58,88]. Carvacrol works on microbial cells, damaging bacterial membranes both structurally and functionally, while magnolol suppresses important bacteria that cause periodontal disease to start [25]. Therefore, reduced oxidative stress following carvacrol and magnolol treatment could be a result, in part, of their antibacterial activity [89] and represents a possible future research direction.

## 5. Conclusions

By significantly lowering blood lipid peroxidation (low MDA values), periodontal gels containing both magnolol and carvacrol showed an antioxidant effect in diabetes-related periodontitis. While statistically not as significant, the combined administration of the two extracts—carvacrol and magnolol—was more effective than magnolol given alone.

## Figures and Tables

**Table 1:** The quantities of substances used for the preparation of hydrogels and their role [35].

Ingredient (Unit of Measure)	The Role in the Formulation	HG1	HG2	HG3	HG4
Carvacrol (g)	Therapeutic agent	0.40	0.40	-	-
Magnolol (mg)	Therapeutic agent	-	-	0.25	0.25
Carbopol 940 (g)	Viscosity increasing agent	1.50	1.00	1.50	1.00
Propylene glycol (g)	Wetting agent	3.00	3.00	3.00	3.00
Ethyl alcohol (g)	Dispersing agent	5.00	5.00	5.00	5.00
Triethanolamine (g)	neutralize	1.00	1.50	1.00	1.50
Double-distilled water (g)	Vehicle	Ad 100.00	Ad 100.00	Ad 100.00	Ad 100.00

HG: hydrogel.

**Table 2:** Carvacrol and magnolol antioxidant activity using the ABTS method.

Antioxidant activity	**ABTS Method**							
	Concentration of carvacrol (mg/mL)	18.75	37.5	75.0	150.0	225.0	300.0	375
	Scavenging activity of carvacrol (%)	23.89 ± 2.01	45.31 ± 2.87	47.89 ± 4.03	54.41 ± 5.41	61.05 ± 5.67	70.49 ± 6.34	80.31 ± 7.24
	Concentration of magnolol (mg/mL)	0.05	0.10	0.20	0.30	0.40	0.50	0.60
	Scavenging activity of magnolol (%)	25.44 ± 2.31	42.16 ± 3.56	70.29 ± 6.11	84.54 ± 7.67	88.41 ± 7.69	90.10 ± 8.74	91.27 ± 7.59

ABTS: 2,2′-azino-bis (3-ethylbenzthiazoline-6-sulphonic acid).

**Table 3:** Carvacrol and magnolol antioxidant activity using the DPPH method.

Antioxidant activity	**DPPH Method**						
	Concentration of carvacrol (mg/mL)	0.037	0.075	0.150	0.225	0.300	0.375
	Inhibition percent of carvacrol (%)	26.89 ± 1.82	35.41 ± 2.40	41.64 ± 3.14	48.88 ± 4.63	60.23 ± 6.11	71.77 ± 8.20
	Concentration of magnolol (mg/mL)	0.010	0.015	0.020	0.025	0.030	0.035
	Inhibition percent of magnolol (%)	39.12 ± 2.44	48.42 ± 4.08	63.38 ± 5.64	70.09 ± 7.01	82.41 ± 6.89	84.54 ± 7.84

DPPH: 2,2-diphenyl-2-picryl-hydrazyl.

**Table 4:** The percentage of carvacrol and magnolol released from hydrogels.

Time/Type of Hydrogel	Carvacrol				Magnolol			
	HG1/PBS	HG1/PBS-Alcohol	HG2/PBS	HG2/PBS-Alcohol	HG3/PBS	HG3/PBS-Alcohol	HG4/PBS	HG4/PBS-Alcohol
0	0	0	0	0	0	0	0	0
5	30.07	41.88	34.54	44.22	23.81	37.69	31.28	41.67
10	41.11	57.99	46.88	59.94	37.97	53.8	43.62	57.39
15	53.22	69.11	58.71	69.16	50.08	64.92	55.45	67.61
30	73.31	81.88	77.19	83.05	70.17	76.69	73.93	79.98
45	79.98	84.22	83.01	85.85	76.84	81.03	79.75	83.81
60	82.37	84.54	85.63	87.41	79.23	81.35	82.37	84.86
75	82.99	85.27	86.28	87.81	79.85	82.08	83.02	85.16
90	83.81	86.34	87.16	88.96	80.67	83.15	83.9	85.41
105	85.46	86.89	88.37	90.58	82.32	83.7	85.11	87.03
120	85.91	87.8	88.52	90.92	82.77	84.61	85.26	87.37

HG: hydrogel; PBS: phosphate tampon.

**Table 5:** ANOVA for MDA and CAT for groups C, D, P and PD in blood.

Variable	Total Sum of Squares	F	*p*
MDA	19.386	21.678	0.000
CAT	399,430.733	37.140	0.000

MDA: malondialdehyde; CAT: catalase; *p*-value.

**Table 6:** Scheffe test for C-D-P-PD batch pairs for MDA and CAT in blood.

Variable		MDA		CAT	
Group		Mean Difference	*p*	Mean Difference	*p*
C	D	−1.50087 *	0.000	261.35514 *	0.000
	P	−1.00078 *	0.006	148.04857 *	0.000
	PD	−1.90150 *	0.000	266.05286 *	0.000
D	C	1.50087 *	0.000	−261.35514 *	0.000
	P	0.50009	0.285	−113.30657 *	0.007
	PD	−0.40063	0.475	4.69771	0.999
P	C	1.00078 *	0.006	−148.04857 *	0.000
	D	−0.50009	0.285	113.30657 *	0.007
	PD	−0.90072 *	0.014	118.00429 *	0.005
PD	C	1.90150 *	0.000	−266.05286 *	0.000
	D	0.40063	0.475	−4.69771	0.999
	P	0.90072 *	0.014	−118.00429 *	0.005

MDA: malondialdehyde; CAT: catalase; C: control group; D: diabetes group; P: periodontitis group; PD: periodontitis associated with diabetes group; * significance mean difference values at the significant level of *p* < 0.05.

**Table 7:** ANOVA for MDA and CAT for PD-PDV-PDC-PDM-PDCM groups in blood.

Variable	Total Sum of Squares	F	*p*
MDA	7.069	10.582	0.000
CAT	89,016.947	2.66	0.052

MDA: malondialdehyde; CAT: catalase; *p*-value.

**Table 8:** Scheffe test for lot pairs PD-PDV-PDC-PDM-PDCM for MDA and CAT in blood.

Variable		MDA		CAT	
Group		Mean Difference	*p*	Mean Difference	*p*
PD	PDV	0.31936	0.469	−12.06900	0.993
	PDC	0.39339	0.263	−18.36429	0.968
	PDM	0.80666 *	0.001	−8.54571	0.998
	PDCM	0.95069 *	0.000	−72.52286	0.105
PDV	PD	−0.31936	0.469	12.06900	0.993
	PDC	0.07403	0.995	−6.29529	0.999
	PDM	0.48730	0.102	3.52329	1.000
	PDCM	0.63133 *	0.017	−60.45386	0.239
PDC	PD	−0.39339	0.263	18.36429	0.968
	PDV	−0.07403	0.995	6.29529	0.999
	PDM	0.41327	0.219	9.81857	0.997
	PDCM	0.55730 *	0.045	−54.15857	0.343
PDM	PD	−0.80666 *	0.001	8.54571	0.998
	PDV	−0.48730	0.102	−3.52329	1.000
	PDC	−0.41327	0.219	−9.81857	0.997
	PDCM	0.14403	0.944	−63.97714	0.191
PDCM	PD	−0.95069 *	0.000	72.52286	0.105
	PDV	−0.63133 *	0.017	60.45386	0.239
	PDC	−0.55730 *	0.045	54.15857	0.343
	PDM	−0.14403	0.944	63.97714	0.191

MDA: malondialdehyde; CAT: catalase; PD: periodontitis associated with diabetes group; PDV: periodontitis associated with diabetes treated with vehicle alone; PDC: periodontitis associated with diabetes treated with carvacrol; PDM: periodontitis associated with diabetes treated with magnolol; PDCM: periodontitis associated with diabetes treated with carvacrol and magnolol. * significance mean difference values at the significant level of *p* < 0.05.

**Table 9:** Paired *t*-Student’s test for C-CV lot pair for blood MDA and CAT variables.

Pair	Variable	Mean	SD	*t*	*p*
C-CV	MDA	−0.18597	0.35157	−1.400	0.211
C-CV	CAT	90.17714	100.01154	2.386	0.054

MDA: malondialdehyde; CAT: catalase; C: control group; CV: control group treated with vehicle alone; SD: standard deviation; *p*-value.

## Data Availability

Data are available from the correspondence author upon reasonable request.

## References

[1] Sanz M., Ceriello A., Buysschaert M., Chapple I., Demmer R.T., Graziani F., Herrera D., Jepsen S., Lione L., Madianos P. (2018). Scientific evidence on the links between periodontal diseases and diabetes: Consensus report and guidelines of the joint workshop on periodontal diseases and diabetes by the International Diabetes Federation and the European Federation of Periodontology. Diabetes Res. Clin. Pract..

[2] Mohamed H.G., Idris S.B., Mustafa M., Ahmed M.F., Åstrøm A.N., Mustafa K., Ibrahim S.O. (2015). Impact of chronic periodontitis on levels of glucoregulatory biomarkers in gingival crevicular fluid of adults with and without type 2 diabetes. PLoS ONE.

[3] Casanova L., Hughes F.J., Preshaw P.M. (2014). Diabetes and periodontal disease: A two-way relationship. Br. Dent. J..

[4] Potra Cicalău G.I., Babeș P.A., Domocoș D., Pogan M. (2021). The assessment of two-way relationship between periodontal diseases and diabetes mellitus. Acta Stomatol. Mar..

[5] Sonnenschein S.K., Meyle J. (2015). Local inflammatory reactions in patients with diabetes and periodontitis. Periodontology 2000.

[6] Seymour G.J., Ford P.J., Cullinan M.P., Leishman S., Yamazaki K. (2007). Relationship between periodontal infections and systemic disease. Clin. Microbiol. Infect..

[7] Pardo A., Signoriello A., Signoretto C., Messina E., Carelli M., Tessari M., De Manna N.D., Rossetti C., Albanese M., Lombardo G. (2021). Detection of Periodontal Pathogens in Oral Samples and Cardiac Specimens in Patients Undergoing Aortic Valve Replacement: A Pilot Study. J. Clin. Med..

[8] Pihlstrom B.L., Michalowicz B.S., Johnson N.W. (2005). Periodontal diseases. Lancet.

[9] Soskolne W.A., Klinger A. (2001). The relationship betweenperiodontal diseases and diabetes: An overview. Ann. Periodontol..

[10] Ira B.L. (2014). Non-periodontal oral complication of diabetes mellitus. Diabetes Mellitus and OralHealth: An Interprofessional Approach.

[11] Stöhr J., Barbaresko J., Neuenschwander M., Schlesinger S. (2021). Bidirectional association between periodontal disease and diabetes mellitus: A systematic review and meta-analysis of cohort studies. Sci. Rep..

[12] Sies H., Berndt C., Jones D.P. (2017). Oxidative stress. Annu. Rev. Biochem..

[13] Nguyen T.T., Ngo L.Q., Promsudthi A., Surarit R. (2016). Salivary oxidative stress biomarkers in chronic periodontitis and acute coronary syndrome. Clin. Oral. Investig..

[14] Halliwell B. (1994). Free radicals, antioxidants, and human disease: Curiosity, cause, or consequence?. Lancet.

[15] Bullon P., Newman H.N., Battino M. (2014). Obesity, diabetes mellitus, atherosclerosis and chronic periodontitis: A shared pathology via oxidative stress and mitochondrial dysfunction?. Periodontology 2000.

[16] Yamamoto Y., Hirose H., Saito I., Nishikai K., Saruta T. (2004). Adiponectin, an adipocyte-derived protein, predicts future insulin resistance: Two-year follow-up study in Japanese population. J. Clin. Endocrinol. Metab..

[17] Borges I., Moreira E.A., Filho D.W., de Oliveira T.B., da Silva M.B., Fröde T.S. (2007). Proinflammatory and oxidative stress markers in patients with periodontal disease. Mediators Inflamm..

[18] Qu H. (2023). The association between oxidative balance score and periodontitis in adults: A population-based study. Front. Nutr..

[19] Gaweł S., Wardas M., Niedworok E., Wardas P. (2004). Malondialdehyde (MDA) as a lipid peroxidation marker. Wiad. Lek..

[20] Ayala A., Muñoz M.F., Argüelles S. (2014). Lipid peroxidation: Production, metabolism, and signaling mechanisms of malondialdehyde and 4-hydroxy-2-nonenal. Oxid. Med. Cell. Longev..

[21] Camkurt M.A., Fındıklı E., Bakacak M., Tolun F.İ., Karaaslan M.F. (2017). Evaluation of Malondialdehyde, Superoxide Dismutase and Catalase Activity in Fetal Cord Blood of Depressed Mothers. Clin. Psychopharmacol. Neurosci..

[22] Bouayed J., Bohn T. (2010). Exogenous antioxidants—Double-edged swords in cellular redox state: Health beneficial effects at physiologic doses versus deleterious effects at high doses. Oxid. Med. Cell. Longev..

[23] Ighodaro O.M., Akinloye O.A. (2018). First line defence antioxidants-superoxide dismutase (SOD), catalase (CAT) and glutathione peroxidase (GPX): Their fundamental role in the entire antioxidant defence grid. Alex. J. Med..

[24] Samarghandian S., Borji A., Farahmand S.K., Afshari R., Davoodi S. (2013). *Crocus sativus* L. (saffron) stigma aqueous extract induces apoptosis in alveolar human lung cancer cells through caspase-dependent pathways activation. BioMed Res. Int..

[25] Cicalău G.I.P., Babes P.A., Calniceanu H., Popa A., Ciavoi G., Iova G.M., Ganea M., Scrobotă I. (2021). Anti-Inflammatory and Antioxidant Properties of Carvacrol and Magnolol, in Periodontal Disease and Diabetes Mellitus. Molecules.

[26] Liang W.Z., Lu C.H. (2012). Carvacrol-induced [Ca^2+^] i rise and apoptosis in human glioblastoma cells. Life Sci..

[27] Samarghandian S., Farkhondeh T., Samini F., Borji A. (2016). Protective effects of carvacrol against oxidative stress induced by chronic stress in rat’s brain, liver, and kidney. Biochem. Res. Int..

[28] Aristatile B., Al-Numair K.S., Al-Assaf A.H., Veeramani C., Pugalendi K.V. (2015). Protective effect of carvacrol on oxidative stress and cellular DNA damage induced by UVB irradiation in human peripheral lymphocytes. J. Biochem. Mol. Toxicol..

[29] Chen H.Y., Hung Y.C., Lee E.J., Chen T.Y., Chuang I.C., Wu T.S. (2009). The protective efficacy of magnolol in hind limb ischemia-reperfusion injury. Phytomedicine.

[30] Li X., Fang Q., Lin J., Yuan Z. (2014). Chemistry Study on Protective Effect against· OH-induced DNA Damage and Antioxidant Mechanism of Cortex Magnoliae Officinalis. Bull. Korean Chem. Soc..

[31] Re R., Pellegrini N., Proteggente A., Pannala A., Yang M., Rice-Evans C. (1999). Antioxidant activity applying an improved ABTS radical cation decolorization assay. Free Radic. Biol. Med..

[32] Kedare S.B., Singh R.P. (2011). Genesis and development of DPPH method of antioxidant assay. J. Food Sci. Technol..

[33] Foti M.C. (2007). Antioxidant properties of phenols. J. Pharm. Pharmacol..

[34] Litwinienko G., Ingold K.U. (2007). Solvent effects on the rates and mechanisms of reaction of phenols with free radicals. Acc. Chem. Res..

[35] Cicalău G.I., Miere F., Mandal A.K., Ganea M., Scrobota I., Ciavoi G., Jurca C.M. (2022). Formulation and Characterization of Hydrophilic Ointment Bases with Carvacrol and Magnolol for Periodontal Application. Pharmacophore.

[36] Salamanca C.H., Barrera-Ocampo A., Lasso J.C., Camacho N., Yarce C.J. (2018). Franz Diffusion Cell Approach for Pre-Formulation Characterisation of Ketoprofen Semi-Solid Dosage Forms. Pharmaceutics.

[37] Langhans S.A. (2018). Three-Dimensional in Vitro Cell Culture Models in Drug Discovery and Drug Repositioning. Front. Pharmacol..

[38] Yamasaki A., Nikai H., Niitani K., Ijuhin N. (1979). Ultrastructure of the junctional epithelium of germfree rat gingiva. J. Periodontol..

[39] Struillou X., Boutigny H., Soueidan A., Layrolle P. (2010). Experimental animal models in periodontology: A review. Open Dent. J..

[40] Guessous F., Huynh C., N’guyen H., Godeau G., Giroud J.P., Meyer J., Hornebeck W., Roch-Arveiller M. (1994). An animal model for the assessment of gingival lesions. J. Pharmacol. Toxicol. Methods.

[41] Eslami B., Behnia H., Javadi H., Khiabani K.S., Saffar A.S. (2003). Histopathologic comparison of normal and hyperplastic condyles. Oral Surg. Oral. Med. Oral Pathol. Oral Radiol. Endod..

[42] Ramesh A., Varghese S.S., Doraiswamy J.N., Malaiappan S. (2016). Herbs as an antioxidant arsenal for periodontal diseases. J. Intercult. Ethnopharmacol..

[43] Govindappa M. (2015). A Review on Role of Plant(s) Extracts and its Phytochemicals for the Management of Diabetes. J. Diabetes Metab..

[44] Moldovan R., Mitrea D.R., Florea A., David L., Mureşan L.E., Chiş I.C., Suciu Ş.M., Moldovan B.E., Lenghel M., Chiriac L.B. (2023). Effects of Gold Nanoparticles Functionalized with *Cornus mas* L. Fruit Extract on the Aorta Wall in Rats with a High-Fat Diet and Experimental-Induced Diabetes Mellitus-An Imaging Study. Nanomaterials.

[45] Karunanayake E.H., Hearse D.J., Mellows G. (1975). The metabolic fate and elimination of streptozocin. Biochem. Soc. Trans..

[46] Akbarzadeh A., Norouzian D., Mehrabi M.R., Jamshidi S., Farhangi A., Verdi A.A., Mofidian S.M., Rad B.L. (2007). Induction of diabetes by Streptozotocin in rats. Indian J. Clin. Biochem..

[47] Iova G.M., Calniceanu H., Popa A., Szuhanek C.A., Marcu O., Ciavoi G., Scrobota I. (2021). The Antioxidant Effect of Curcumin and Rutin on Oxidative Stress Biomarkers in Experimentally Induced Periodontitis in Hyperglycemic Wistar Rats. Molecules.

[48] Akbarzadeh A., Noruzian D., Jamshidi S., Farhangi A., Mehrabi M.R., Rad B.L., Mofidian M., Allahverdi A. (2007). Treatment of streptozotocin induced diabetes in male rats by immunoisolated transplantation of islet cells. Indian J. Clin. Biochem..

[49] Vargas-Sanchez P.K., Moro M.G., Santos F.A.D., Anbinder A.L., Kreich E., Moraes R.M., Padilha L., Kusiak C., Scomparin D.X., Franco G.C.N. (2017). Agreement, correlation, and kinetics of the alveolar bone-loss measurement methodologies in a ligature-induced periodontitis animal model. J. Appl. Oral. Sci..

[50] Ribeiro D.D.S.F., Freire J.M.O., Teixeira A.H., Val D.R.D., Freitas A.R., Gomes F.I.F., Silva A.A.R.E., Bandeira P.N., Santos H.S.D., Santos W.P.D. (2018). Tocoyena sellowiana extract decreases bone loss in an experimental model of periodontitis in rats: Putative role for cyclooxygenase-2 and IL-1? inhibition. Biomed. Pharmacother..

[51] Teixeira A.H., Freire J.M.O., de Sousa L.H.T., Parente A.T., de Sousa N.A., Arriaga A.M.C., Lopes da Silva F.R., Melo I.M., Castro da Silva I.I., Pereira K.M.A. (2017). *Stemodia maritima* L. Extract Decreases Inflammation, Oxidative Stress, and Alveolar Bone Loss in an Experimental Periodontitis Rat Model. Front. Physiol..

[52] Marins L.M., Napimoga M.H., Malta F.S., Miranda T.S., Nani E.P., Franco B.D.S.T., da Silva H.D.P., Duarte P.M. (2020). Effects of strontium ranelate on ligature-induced periodontitis in estrogen-deficient and estrogen-sufficient rats. J. Periodontal. Res..

[53] Hosadurga R.R., Rao S.N., Jose J., Rompicharla N.C., Shakil M., Shashidhara R. (2014). Evaluation of the efficacy of 2% curcumin gel in the treatment of experimental periodontitis. Pharmacogn. Res..

[54] Duarte P.M., Tezolin K.R., Figueiredo L.C., Feres M., Bastos M.F. (2010). Microbial profile of ligature-induced periodontitis in rats. Arch. Oral Biol..

[55] Wang T.H., Hsia S.M., Wu C.H., Ko S.Y., Chen M.Y., Shih Y.H., Shieh T.M., Chuang L.C., Wu C.Y. (2016). Evaluation of the antibacterial potential of liquid and vapor phase phenolic essential oil compounds against oral microorganisms. PLoS ONE.

[56] Maquera-Huacho P.M., Tonon C.C., Correia M.F., Francisconi R.S., Bordini E.A.F., Marcantonio É., Spolidorio D.M.P. (2018). In vitro antibacterial and cytotoxic activities of carvacrol and terpinen-4-ol against biofilm formation on titanium implant surfaces. Biofouling.

[57] Ho K.Y., Tsai C.C., Chen C.P., Huang J.S., Lin C.C. (2001). Antimicrobial activity of honokiol and magnolol isolated from Magnolia officinalis. Phytother. Res..

[58] Saito J., Sakai Y., Nagase H. (2006). In vitro anti-mutagenic effect of magnolol against direct and indirect mutagens. Mutat. Res..

[59] Kohlert C., Schindler G., März R.W., Abel G., Brinkhaus B., Derendorf H., Gräfe E.U., Veit M. (2002). Systemic availability and pharmacokinetics of thymol in humans. J. Clin. Pharm..

[60] Conti M., Morand P.C., Levillain P., Lemonnier A. (1991). Improved fluorometric determination of malonaldehyde. Clin. Chem..

[61] Pippenger C.E., Browne R.W., Armstrong D. (1998). Regulatory antioxidant enzymes. Methods Mol. Biol..

[62] Erejuwa O.O., Sulaiman S.A., Wahab M.S.A., Sirajudeen K.N.S., Salleh M.S.M., Gurtu S. (2010). Antioxidant Protective Effect of Glibenclamide and Metformin in Combination with Honey in Pancreas of Streptozotocin-Induced Diabetic Rats. Int. J. Mol. Sci..

[63] Bajaj S., Khan A. (2012). Antioxidants and diabetes. Indian J. Endocrinol. Metab..

[64] Mansouri E., Panahi M., Ghaffari M.A., Ghorbani A. (2011). Effects of grape seed proanthocyanidin extract on oxidative stress induced by diabetes in rat kidney. Iran. Biomed. J..

[65] Mastelić J., Jerković I., Blažević I., Poljak-Blaži M., Borović S., Ivančić-Baće I., Smrečki V., Žarković N., Brčić-Kostic K., Vikić-Topić D. (2008). Comparative study on the antioxidant and biological activities of carvacrol, thymol, and eugenol derivatives. J. Agric. Food Chem..

[66] Kumar D., Rawat D.S. (2013). Synthesis and antioxidant activity of thymol and carvacrol based Schiff bases. Bioorganic Med. Chem. Lett..

[67] Tan L.H., Zhang D., Yu B., Zhao S.P., Cao W.G. (2015). Antioxidant activity of the different polar solvent extracts of Magnolia officinalis leaves and purification of main active compounds. Eur. Food Res. Technol..

[68] Llana-Ruiz-Cabello M., Gutiérrez-Praena D., Puerto M., Pichardo S., Jos Á., Cameán A.M. (2015). In vitro pro-oxidant/antioxidant role of carvacrol, thymol and their mixture in the intestinal Caco-2 cell line. Toxicol. In Vitro.

[69] de Molon R.S., de Avila E.D., Cirelli J.A. (2013). Host responses induced by different animal models of periodontal disease: A literature review. J. Investig. Clin. Dent..

[70] Goyal S.N., Reddy N.M., Patil K.R., Nakhate K.T., Ojha S., Patil C.R., Agrawal Y.O. (2016). Challenges and issues with streptozotocin-induced diabetes—A clinically relevant animal model to understand the diabetes pathogenesis and evaluate therapeutics. Chem. Biol. Interact..

[71] Draper H.H., Hadley M. (1990). Malondialdehyde determination as index of lipid peroxidation. Methods Enzymol..

[72] Padalkar R.K., Shinde A.V., Patil S.M. (2012). Lipid profile, serum malondialdehyde, superoxide dismutase in chronic kidney diseases and Type 2 diabetes mellitus. Biomed. Res..

[73] De Araújo R.F., Souza T.O., De Moura L.M., Torres K.P., De Souza L.B., Alves M.D.S.C.F., Rocha H.O., De Araújo A.A. (2013). Atorvastatin Decreases Bone Loss, Inflammation and Oxidative Stress in Experimental Periodontitis. PLoS ONE.

[74] Oktay S., Chukkapalli S.S., Rivera-Kweh M.F., Velsko I.M., Holliday L.S., Kesavalu L. (2015). Periodontitis in rats induces systemic oxidative stress that is controlled by bone-targeted antiresorptives. J. Periodontol..

[75] Li X., Sun X., Zhang X., Mao Y., Ji Y., Shi L., Cai W., Wang P., Wu G., Gan X. (2018). Enhanced Oxidative Damage and Nrf2 Downregulation Contribute to the Aggravation of Periodontitis by Diabetes Mellitus. Oxid. Med. Cell. Longev..

[76] Guimarães A.G., Oliveira G.F., Melo M.S., Cavalcanti S.C., Antoniolli A.R., Bonjardim L.R., Silva F.A., Santos J.P., Rocha R.F., Moreira J.C. (2010). Bioassay-guided evaluation of antioxidant and antinociceptive activities of carvacrol. Basic. Clin. Pharm. Toxicol..

[77] Aeschbach R., Löliger J., Scott B.C., Murcia A., Butler J., Halliwell B., Aruoma O.I. (1994). Antioxidant actions of thymol, carvacrol, 6-gingerol, zingerone and hydroxytyrosol. Food Chem. Toxicol..

[78] Aydın E., Türkez H., Keleş M.S. (2014). The effect of carvacrol on healthy neurons and N2a cancer cells:Some biochemical, anticancerogenicity and genotoxicity studies. Cytotechnology.

[79] Hariri A.T., Moallem S.A., Mahmoudi M., Memar B., Hosseinzadeh H. (2010). Sub-acute effects of diazinon on biochemical indices and specific biomarkers in rats: Protective effects of crocin and safranal. Food Chem. Toxicol..

[80] Kohen R., Nyska A. (2002). Oxidation of biological systems: Oxidative stress phenomena, antioxidants, redox reactions, and methods for their quantification. Toxicol. Pathol..

[81] de Carvalho F.O., Silva É.R., Gomes I.A., Santana H.S.R., do Nascimento Santos D., de Oliveira Souza G.P., de Jesus Silva D., Monteiro J.C.M., de Albuquerque Júnior R.L.C., de Souza Araújo A.A. (2020). Anti-inflammatory and antioxidant activity of carvacrol in the respiratory system: A systematic review and meta-analysis. Phytother. Res..

[82] Tabibzadeh Dezfuli S.A., Ehsani M., Lakzaei Azar O. (2017). Carvacrol Alleivated Negative Effects of Diabetes on Inflammation and Oxidation by Modulation in Gene Expression of Inflammatory and Antioxidant System in Diabetic Rat Model. GMJ Med..

[83] Abiko Y., Selimovic D. (2010). The mechanism of protracted wound healing on oral mucosa in diabetes. Review. Bosn. J. Basic. Med. Sci..

[84] Lalla E., Lamster I.B., Stern D.M., Schmidt A.M. (2001). Receptor for advanced glycation end products, inflammation, and accelerated periodontal disease in diabetes: Mechanisms and insights into therapeutic modalities. Ann. Periodontol..

[85] Yamagishi S., Maeda S., Matsui T., Ueda S., Fukami K., Okuda S. (2012). Role of advanced glycation end products (AGEs) and oxidative stress in vascular complications in diabetes. Biochim. Biophys. Acta.

[86] Wang J.J., Zhao R., Liang J.C., Chen Y. (2014). The antidiabetic and hepatoprotective effects of magnolol on diabetic rats induced by high-fat diet and streptozotocin. Yao Xue Xue Bao.

[87] Liu C.M., Chen S.H., Liao Y.W., Yu C.H., Yu C.C., Hsieh P.L. (2021). Magnolol ameliorates the accumulation of reactive oxidative stress and inflammation in diabetic periodontitis. J. Med. Assoc..

[88] Botelho M.A., Rao V.S., Montenegro D., Bandeira M.A., Fonseca S.G., Nogueira N.A., Ribeiro R.A., Brito G.A. (2008). Effects of a herbal gel containing carvacrol and chalcones on alveolar bone resorption in rats on experimental periodontitis. Phytother. Res..

[89] Tóthová L., Celec P. (2017). Oxidative Stress and Antioxidants in the Diagnosis and Therapy of Periodontitis. Front. Physiol..

